# Improving the reproducibility and integrity of research: what can different stakeholders contribute?

**DOI:** 10.1186/s13104-022-06030-2

**Published:** 2022-04-25

**Authors:** Malcolm Macleod, the University of Edinburgh Research Strategy Group

**Affiliations:** Academic Lead for Research Improvement and Research Integrity, University of Edinburgh, Charles Stewart House, Chambers Street, Edinburgh, Scotland

**Keywords:** Research integrity, Researcher integrity, Research improvement, Research reproducibility

## Abstract

Increasing awareness of problems with the reproducibility and integrity of research led the UK Parliament Science and Technology Committee to launch, in July 2021, an inquiry into reproducibility and research integrity. We recognise at least four potential reasons why attempts to replicate a research finding may be unsuccessful: false positive statistical analyses, low generalisability of findings, suboptimal study designs (research integrity), and deliberate malfeasance (researcher integrity). It is important to make a distinction between the contributions of research integrity and of researcher integrity to the reproducibility crisis. While the impact of an individual instance of compromised researcher integrity is substantial, the aggregate impact of more prevalent problems with research integrity is likely much greater. The research community will be most efficient when failed replication efforts are never due to issues of research integrity or of researcher integrity, as this would allow focus on the scientific reasons for why two apparently similar experiments should reach different conclusions. We discuss the role of funders, institutions and government in addressing the “reproducibility crisis” before considering which interventions might have a positive impact on academia’s approach to reproducible research, and a possible role for a committee on research integrity.

## Introduction

Science is held to be “self-correcting”, in that erroneous findings are identified in subsequent research, and our understanding of the underlying phenomena is thereby revised. It has been argued [1] that this process might take one of two forms—diagnostic replication (“*concerned with evaluating the truth value of a claim*”) and integrative replication (“*concerned with incorporating findings from a study for one’s own purposes”*). Self correction may arise from either process, but replication failure is more obvious with the first. Indeed, several diagnostic replication projects (for instance in psychology [2] and in cancer biology [3]) have shown that at least half of reported findings could not be replicated. In July 2021 the UK Parliament Science and Technology Committee initiated an Inquiry into reproducibility and research integrity, soliciting written evidence relating to the breadth, causes, and mitigation of this “reproducibility crisis”. Here we summarise the submission from the University of Edinburgh. As a research performing organisation, our submission had greatest focus on potential actions relevant to this role.

## Main text

### Causes

Our primary concern as a research institution is that the findings of our research should be useful to research users. Where we or others have sought and failed to replicate the findings of our own research or that of others, we recognise at least four potential reasons for this (Table 1).

**Table 1:** Categories of reasons why attempts to replicate a research finding may be unsuccessful

Category 1	A valid research claim was made based on the observed data, but the statistical test had returned a Type I or “false positive” error
Category 2	The claim that was made was valid under the particular circumstances under which it was tested but is not observed under the circumstance in which replication was attempted. These different circumstances may be obvious or subtle, and their impact on the observed phenomena may or may not be important in understanding the question at hand
Category 3	The observations may have been due to sub-optimal study designs (which for instance allow the emergence of experimenter bias, or selective data presentation, or hypothesising after results are known), which might generally be considered as questionable research practices, with varying degrees of researcher culpability
Category 4	The research claim may have been made following deliberate researcher malfeasance such as falsification or fabrication

In our view the first and second explanations—Type I statistical errors and a failure to generalise [4]—cannot be characterised as part of a “reproducibility crisis” because they are integral to the scientific process. They only cause problems where research findings become part of a canon of belief without appropriate scrutiny or replication, and where our research ecosystems discourage the funding, conduct and publication of replication studies and of neutral results.

The third explanation—of suboptimal research practices—can be characterised as contributing to a crisis in reproducibility in that it relates to harms which are avoidable. There is broad consensus that the research ecosystem (and, in particular, systems of funding, publication and promotion) conditions researchers to behave in ways which maximise the prospects for “success” in these narrow terms but has adverse consequences in the reliability of research outputs [5]. We do not consider this a deliberate manipulation or subversion of the research process. Importantly, identification of these practices provides direction for research improvement based on training, audit, and the provision of resources and incentive structures which enable and encourage researchers to do their best work [6]. This third category relates to the integrity of a research claim.

In contrast, deliberate researcher malfeasance (the fourth explanation) is completely unacceptable. This fourth category relates to the integrity with which the researcher conducted the research. It is we believe critical to make a distinction between the contributions of research integrity (category 3) and of researcher integrity (category 4) to the reproducibility crisis. While the impact of an individual instance of compromised researcher integrity is substantial, the aggregate impact of more prevalent problems with research integrity is likely much greater [7].

We appreciate that some readers will not agree with such a clear distinction between research integrity and researcher integrity. It has been argued in review that both of these are products of researcher behaviour, and that they have a fluid and inter-dependent relationship. Indeed, we would agree that a researcher who continues to pursue suboptimal research practices despite knowing that these are suboptimal demonstrates compromised researcher integrity. However, we find the distinction to be helpful in our institutional efforts in research improvement, as it facilitates discussions in research integrity without the implicit accusation that our target audience are not acting with professional integrity.

Our research endeavours will be most efficient when failed replication efforts are never due to issues of research integrity or of researcher integrity, as this would allow focus on the scientific reasons for why two apparently similar experiments should reach different conclusions. Importantly, concerns about reproducibility and research integrity feed into wider issues of public trust in science and in the policy which this informs. It is therefore crucial that we ensure a well-founded credibility and authority for science in public debates and amongst policy makers, as they seek to apply and exploit research findings for the public good.

### The role of funders in addressing the “reproducibility crisis”

The drivers of the behaviours and research approaches which lead to poor reproducibility, and the prevalence of such problems and their consequences for the progress of science, are well described (see for instance [5, 8]). However, less is known of the effectiveness of interventions to improve research reproducibility. Many solutions have been proposed but few have been subjected to rigorous tests of whether they work as expected. Where they have been tested, interventions may be without effect (see for instance [9]).

In our view, the generation of evidence for what works in research improvement is an important and legitimate topic for research funding. While there are some funding schemes relevant to aspects of such work, they do not have a specific remit for developing and testing interventions in research improvement. Further, a three-year funding cycle discourages the development of interventions which may require several years to show a benefit. These are missed opportunities, and provision of ring-fenced funding for “research on research” is desirable.

Replication studies provide exceptional value for money, in that they have a high probability of revising existing knowledge. An efficient research funding model would contain a substantial stream dedicated to prospective replication studies. Within a given research field, the establishment of multicentre consortia to conduct such research would also—by emphasising the importance of preregistration and rigorous experimental design—have beneficial effects on other (non-replication) research conducted by consortium members.

### The role of Research institutions in addressing the “reproducibility crisis”

Recent years have seen increasingly detailed discussion of the role of research performing institutions in promoting research integrity [10, 11], much of which is relevant to research reproducibility. Institutions should have robust systems in place to identify issues of researcher integrity, to respond to internal or external allegations of such activity, and to take appropriate action. However, we believe a more important role is in engendering research integrity (category 3 above). Appropriate researcher behaviours might be encouraged by ensuring they have the capabilities to do excellent research, through education, training and mentoring; opportunities to do so, for instance through the provision of tools to support open publication, data sharing and pre-registration; and motivation, for instance through appointment and promotion criteria which are more concerned with research quality and less concerned with where it was published or how much grant income was generated, and which recognise broader contributions to good research citizenship [12]. This should be accompanied by greater attention to continuing professional development for researchers, including time for reflection on the strengths and weaknesses of their current research approaches, and the development of skills in research improvement approaches.

Of course, the behaviours of researchers and research institutions are deeply ingrained and shaped by external as well as internal forces. For instance, while a PhD student may be entirely confident that their own institution recognises and rewards excellence in research processes, if they do not have this confidence in other institutions where they might seek future employment, they may feel obliged to seek more conventional markers of esteem. Enabling a “levelling up” across UK institutions—for instance through organisations such as the UK Reproducibility Network [13]—will help address this concern.

Further, we believe that there may be substantial opportunities in applying improvement methods—widespread in healthcare, industry and even sporting endeavour—to the process of producing research [14]. First, a research group asserts what they consider to represent best practice, based for instance on external guidelines for the conduct and reporting of research, on the findings of research on research, or on the expressed needs of research users. Current performance is evaluated and strategies developed to improve that performance. These are then piloted in small tests of change. If performance improves, we adopt the change, and if not, we revise the strategy and try again. Through focussing on delivering improvements in the way we do research, we seek to embed a system of “quality by design”, to complement the external “quality by outputs evaluation” involved for instance in the Research Excellence Framework [15].

### What might have a positive impact on academia’s approach to reproducible research?

In essence, we need to value the quality of the research process more than we do the results of that research. For us this means.

- Richer and deeper training and education in rigorous research practices.
- Changes to criteria for appointment and promotion to value researcher behaviours as well as researcher outputs.
- Institutional investment in quality by design.
- Emphasising professional obligations of researchers in good research citizenship.
- Seeking to embed best practice as a habit rather than a rigid straight-jacket.

A key insight is that issues of reproducibility—what one might consider as the provenance of a research claim- are likely to be problematic in most disciplines—and we should proceed on the basis that they are present unless we can demonstrate that they are not.

Finally, the precarious nature of research careers is such that early career researchers, and their supervisors, spend much of their time focussing on where they will get their next three years of funding—or what they will do if they do not. This continual pressure is one reason for the drive to publication, even if the work is not quite ready and the findings are not quite secure. Re-shaping research careers to reduce the metronomic requirement for stellar results would go some way to improving research practice. Of several options, the provision of “run through training” from first post-doc to independence, with funding secured for 7–10 years, might be helpful. Another approach would be to shift the balance of salary source, so that a higher proportion came in core funding and less came through research grants. These are complex issues, and a national review of research career pathways may be helpful.

Institutions seeking to implement change will wish to have confidence that that change will be effective, and will provide good value. Taking for example the provision of enhanced statistical and methodological support, while there is some consensus that this would be “a good thing”, we do not know by how much it might improve performance or what the costs might be. In such circumstances, testing interventions in randomised trials may be helpful.

**Fig. 1:**
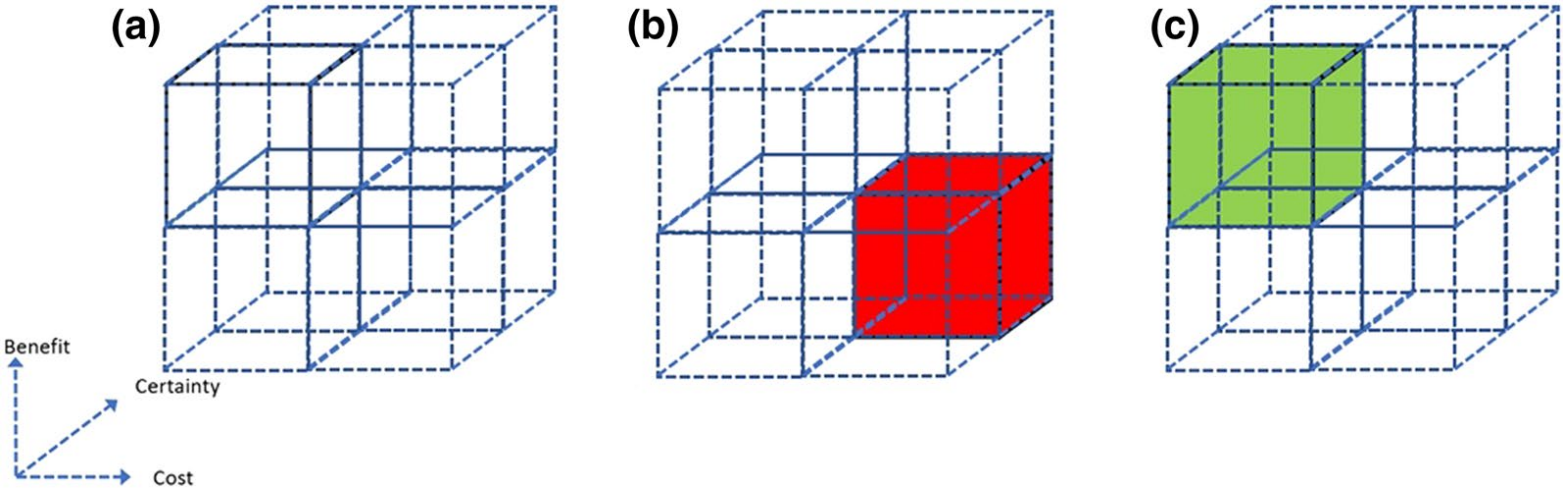
The research improvement cube. **a** The costs (x axis), potential benefits (y axis) and our certainty in these estimates (z axis) can be portrayed in three dimensional space. **b** An intervention which is known, with confidence, to have high cost and low benefit is unlikely to be implemented. **c** An intervention for which there is low certainty in costs of benefits, but a suggestion of low cost and high benefit, might be suitable for implementation with audit to establish if the expected changes occur

Proposed interventions can be mapped in three dimensional space according to cost, potential benefit, and the certainty in these estimates (Fig. 1). Where there is certainty, implementation decisions can be informed by institutional prioritisation, but where there is uncertainty, options include implementation with audit (where costs are low) or randomised studies.

### The role of Government in addressing the “reproducibility crisis”

We expect the governments will seek to maximise the value of taxpayer investment in research. This value comes not just from research outputs, but also from closer interactions between academia and industry; the economic contribution of start-up and spin-out companies; and the availability of a skilled research workforce available for recruitment to the private sector. To maximise this value, we therefore need to focus not only on the reliability, credibility, and reproducibility of research outputs, but also on ensuring that our research workforce is well versed in the approaches to increasing reproducibility described herein. It would be surprising if government did not seek to ensure that intermediate agencies such as UKRI sought to maximise the reproducibility of the work which they funded.

### Would establishing a national committee on research integrity under UKRI impact the reproducibility crisis?

Research integrity is an issue in every aspect of the work of UKRI, and there is a risk that the establishment of a national committee could lead other parts of the organisation to think that responsibility for research integrity did not lie with them. It is also critically important that UKRI makes a distinction between research integrity and researcher integrity. The role of such a committee should include (i) an annual audit of UKRIs efforts to support research integrity; (ii) funding research in and pilot implementation of research integrity improvement projects; and (iii) developing proposals for how an institution’s systems to support research improvement can be recognised in UKRI funding decisions and in research evaluation exercises, rewarding systems which provide “quality by design”.

### Outlook

It is right that the Science and Technology Committee of the UK Parliament is concerned with the research reproducibility. Efforts in research improvement, led from the research community, have the potential to cement the UKs reputation as a global research power. If we do not engage, wholeheartedly, in such efforts, it is possible that more formal systems of research oversight may be deemed necessary.

## Data Availability

Not applicable.

## Abbreviations

STC: UK Parliament Science and Technology Committee
REF: Research Excellence Framework
UKRI MRC: UKRI Medical Research Council
UKRI: UK Research and Innovation
